# Metabolomic Profiling of Blood-Derived Microvesicles in Breast Cancer Patients

**DOI:** 10.3390/ijms222413540

**Published:** 2021-12-17

**Authors:** Judith Buentzel, Henry Gerd Klemp, Ralph Kraetzner, Matthias Schulz, Gry Helene Dihazi, Frank Streit, Annalen Bleckmann, Kerstin Menck, Darius Wlochowitz, Claudia Binder

**Affiliations:** 1 Department of Hematology and Medical Oncology, University Medical Center Goettingen, 37075 Goettingen, Germany; mschulz@gdwg.de (M.S.); annalen.bleckmann@ukmuenster.de (A.B.); claudia.binder@med.uni-goettingen.de (C.B.); 2 Department of Pediatrics and Adolescent Medicine, University Medical Center Goettingen, 37075 Goettingen, Germany; henrygerd.klemp@stud.uni-goettingen.de (H.G.K.); rkraetzner@gwdg.de (R.K.); 3 Metabolomics Platform, Department of Clinical Chemistry, University Medical Center Goettingen, 37075 Goettingen, Germany; gryhelene.dihazi@med.uni-goettingen.de (G.H.D.); frank.streit@med.uni-goettingen.de (F.S.); 4 Department of Medicine A (Hematology, Oncology, Hemostaseology and Pulmonology), University Hospital Muenster, 48149 Muenster, Germany; kerstin.menck@ukmuenster.de; 5 Medical Bioinformatics, University Medical Center Goettingen, 37075 Goettingen, Germany; dwl@bioinf.med.uni-goettingen.de

**Keywords:** metabolic profiling, breast cancer, extracellular vesicles, biomarker, molecular breast cancer subtypes

## Abstract

Malignant cells differ from benign ones in their metabolome and it is largely unknown whether this difference is reflected in the metabolic profile of their microvesicles (MV), which are secreted into the blood of cancer patients. Here, they are present together with MV from the various blood and endothelial cells. Harvesting MV from 78 breast cancer patients (BC) and 30 controls, we characterized the whole blood MV metabolome using targeted and untargeted mass spectrometry. Especially (lyso)-phosphatidylcholines and sphingomyelins were detected in a relevant abundance. Eight metabolites showed a significant discriminatory power between BC and controls. High concentrations of lysoPCaC26:0 and PCaaC38:5 were associated with shorter overall survival. Comparing BC subtype-specific metabolome profiles, 24 metabolites were differentially expressed between luminal A and luminal B. Pathway analysis revealed alterations in the glycerophospholipid metabolism for the whole cancer cohort and in the ether lipid metabolism for the molecular subtype luminal B. Although this mixture of blood-derived MV contains only a minor number of tumor MV, a combination of metabolites was identified that distinguished between BC and controls as well as between molecular subtypes, and was predictive for overall survival. This suggests that these metabolites represent promising biomarkers and, moreover, that they may be functionally relevant for tumor progression.

## 1. Introduction

The communication between cancer cells and their microenvironment is an essential mechanism supporting tumor progression, invasion and metastasis. This crosstalk is mediated in part by secreted soluble messengers but also by extracellular vesicles (EV), which are produced by tumor cells in high numbers. EV have recently attracted increasing attention since they are able to regulate dynamic crosstalk between cancerous, immune and stromal cells in establishing the tumorigenic microenvironment [1]. Apart from apoptotic bodies (500–4000 nm), which represent products of dying cells, there are two main types of EV: microvesicles (MV, 100–1000 nm), which are shed directly from the outer plasma membrane, as well as the small EV (sEV, also called exosomes, 50–150 nm), which are of endosomal origin [2,3,4]. Both MV and sEV carry not only DNA, mRNA or miRNA, but also cytosolic and membrane proteins originating from the mother cell. This cargo can then be transferred to the recipient cells and modulate their biological characteristics [1,5]. Detection of EV in nearly all biological fluids, such as blood, urine or saliva, demonstrates not only the advantage of isolating them from various bodily fluids, but also indicates their crucial role supporting malignant invasion, immune escape, angiogenesis, creating a hospital environment to support cancer growth and prompting therapy escape [6,7].

MV can easily be harvested and analyzed from cancer patients’ blood due to their large size. They can be collected without the need for ultracentrifugation and can subsequently be characterized by flow cytometry. The blood-derived “microvesiculome” mostly comprises MV originating from blood and endothelial cells, while only a minor part or percentage of these MV is tumor derived [8]. Despite this fact, we have shown that cancer patients’ MV strongly induce tumor invasiveness in vitro, while MV from healthy controls do not [8]. This evaluation of MV as a biomarker has recently aroused broader scientific interest.

Until now, the potential of EV as biomarkers in breast cancer patients has mainly been analyzed in small patient cohorts with regard to the proteome and miRNA content of sEV [9,10]. However, many of the EV effects can only partially be explained by pro-invasive proteins or (mi)RNAs. EV also contain lipids and other small metabolites, such as sphingomyelin and ceramide signaling, known to be involved in pro-invasive signaling cascades. So far, only few studies have focused on the metabolomic profiling of body fluids and cancer tissue. In breast cancer patients, specific features in the lipid composition have been demonstrated for blood plasma [11,12], tissue [13,14,15], saliva [16] or urine [17]. Only two studies concentrated on the metabolome of purified EV [18,19]; however, these were isolated from breast cancer cell lines and not from patients’ blood. Nishida-Aoki et al. investigated the metabolite differences of sEV in two triple-negative breast cancer cell lines and found a higher diacylglycerol content in the more metastatic cell line [18]. Roberg-Larsen et al. described an enrichment of 27-hydroxycholesterol in sEV from estrogen-receptor-positive cells [19]. The metabolome of sEV has also been analyzed in urine or tissue of prostate [20,21,22] or blood plasma of lung [23] and pancreatic [24] cancer patients. Together, these preliminary data clearly support the hypothesis that the metabolome of EV mirrors the biological characteristics of the tumor cells and may yield biomarkers to predict and follow the clinical course of the disease.

Since we have shown that MV can be easily and reliably extracted from blood plasma of cancer patients [25], we set out to characterize the metabolomic profile of blood-derived MV in a cohort of 78 breast cancer patients and 30 controls with complete clinical annotation. Using mass spectrometry and a targeted metabolomics approach, we found that a combination of metabolites discriminates between breast cancer patients and healthy controls as well as between breast cancer molecular subtypes and, additionally, is prognostic regarding overall survival.

## 2. Results

### 2.1. Microvesicle Harvest and Characterization

**Figure 1:**
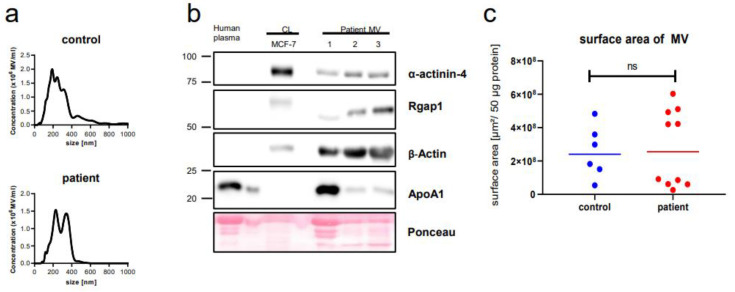
Characterization of plasma microvesicles (MV). MV of the controls and breast cancer patients show a similar concentration and distribution in size (**a**); (**b**) representative Western blot of three independent MV patient samples for common vesicle markers; and (**c**) MV of patients and controls show a similar surface area per 50 µg protein: mean surface was 2.77 × 10^8^ µm^2^ (SD ± 2.30 × 10^8^ µm^2^) and of 2.55 × 10^8^ µm^2^ (SD ± 1.56 × 10^8^ µm^2^) for patients and healthy controls, respectively. CL = whole cell lysate from MCF-7 cells.

The recruited patients and controls are described in the “Section 4” and in Table 1. Nanoparticle tracking analysis of the purified vesicles detected particles with the typical size of MV, ranging from 200 nm to 500 nm, without any difference between patients and controls (Figure 1a).

**Figure A1:**
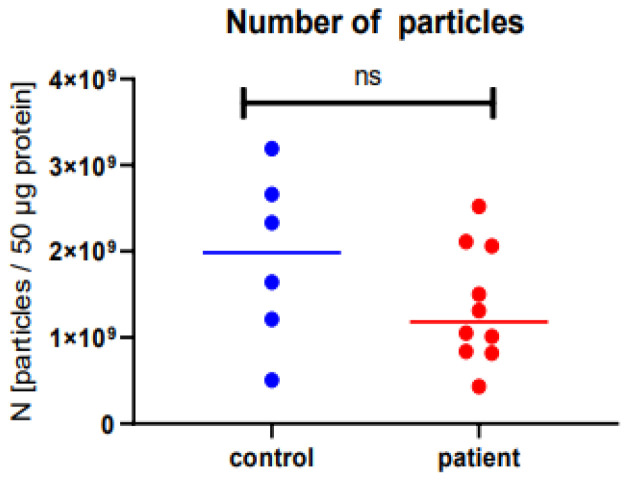
Mean number of MVs standardized to 50 µg protein did not differ significantly between patients (1.37 × 10^9^; SD ± 6.37 × 10^8^) and controls (1.92 × 10^9^; SD ± 9.93 × 10^8^).

Consistently, upon Western blotting the vesicles were positive for markers typically enriched in MV, such as beta-Actin and Rgap1 (Figure 1b). Since it has been shown that plasma lipoproteins can be co-purified with EV, mostly those of small (<100 nm) but also of larger size as in our case [26], we additionally stained for apolipoprotein A1. This contaminant was only detected at higher levels in a minority of cases. In our previous studies, we had established the protein content of the MV preparations as a reliable reference and surrogate marker for the actual MV concentration [25]. Additionally, we did not detect any differences in the number of particles between cancer patients and controls. Two factors could potentially interfere with our metabolomic results: variation in particle numbers and the MV surface area, which corresponds to the lipid-rich plasma membrane. Both particle number and surface were validated again in the present study. In relation to 50 µg of protein, there was no significant difference between the cancer samples and controls (Figure 1c and Figure A1).

### 2.2. Metabolomics by Mass Spectrometry Is Feasible in Blood-Derived Microvesicles

Using the Biocrates AbsoluteIDQ^®^ p180 kit (Innsbruck, Austria), we first applied a targeted metabolomics approach to analyze the metabolome of MV from the blood of controls and breast cancer patients. Although generally the kit can also detect amino acids, biogenic amines, acylcarnitines, glycerophospholipids and sphingomyelins (SM), we only measured the relevant concentrations of the latter two substance classes in our samples (detected metabolites are listed in Table A1). We validated the detection of these lipid classes in parallel for 18 of the samples (13 patients, 5 controls) with an untargeted metabolomics approach. The sample preparation was identical for both methods. Again, we detected mainly lysophosphatidylcholines (lysoPC) and SM as already encountered in the targeted approach. Metabolites detected by both methods are listed in Table 2. While the targeted approach limits the number of potentially detectable metabolites to those included in the kit, the advantage in contrast to the untargeted “open” method was its stability and reproducibility. Therefore, we mainly used this approach for further analyses. Taken together, here we demonstrate the feasibility of targeted as well as untargeted mass spectrometry to analyze the lipidome of blood-derived MV.

### 2.3. Targeted Mass Spectrometry Reveals Differences in the Whole MV Metabolome of Breast Cancer Patients and Controls

**Figure 2:**
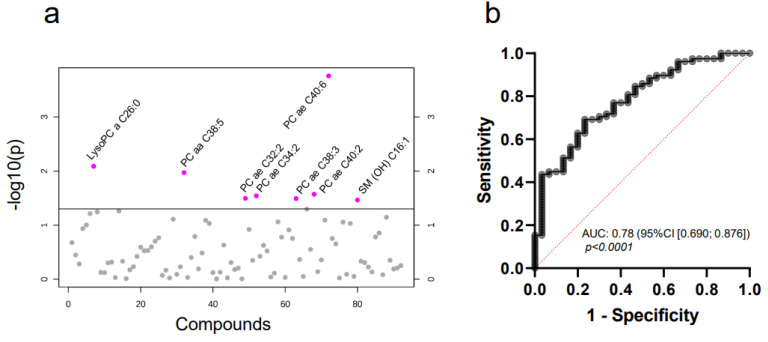
MV-based metabolomics allows the differentiation between healthy controls and breast cancer patients in blood plasma. T-statistics (**a**) reveal eight significant metabolites (*p* ≤ 0.05, two-tailed unpaired Student’s t-test, relevant metabolites are colored in pink) and multiple logistic regression (**b**) for combined Receiver operator characteristic (ROC) analysis of PC ae C40:6, lysoPC a C26:0, PC aa C38:5, PC ae C34:2, PC ae C32:2, PC ae 38:3 and SM (OH) C16:1.

**Figure A2:**
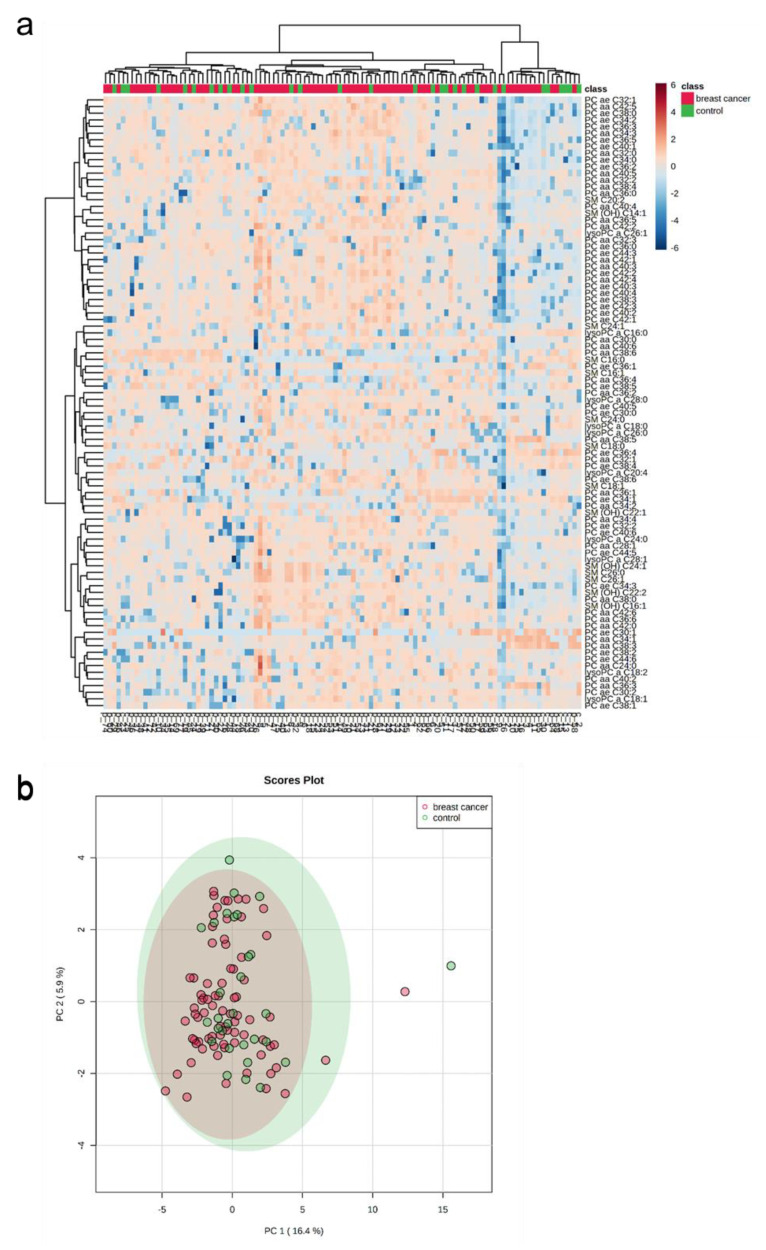
Metabolic profiles of controls and breast cancer patients show no difference in (**a**) the heatmap cluster or (**b**) principal component analysis.

**Figure A3:**
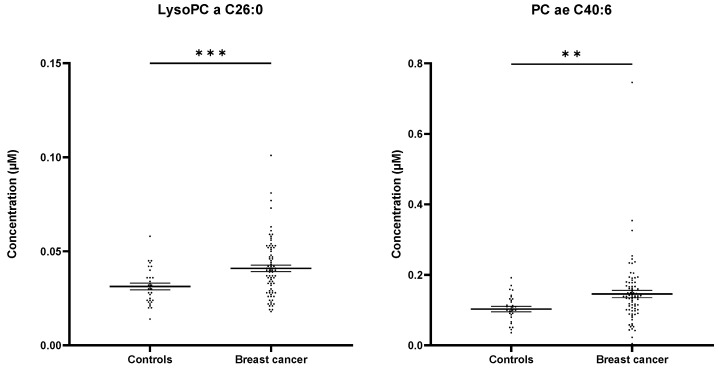
Very-long-chain LysoPC a 26:0 and ether-lipid PC ae 40:6 are elevated in breast cancer patient microvesicles. Concentration of lipids in microvesicles were determined with a p180-kit and displayed using the mean ± SEM. Groups were compared using two-tailed Student’s t-test with the Welsch correction for unequal standard deviations used. **: *p* < 0.01; ***: *p* < 0.001.

Next, we asked whether the metabolome of MV from breast cancer patients differs from that of healthy controls. Interestingly, the first steps in analyzing the data by both PCA and heatmapping yielded no major difference in the general metabolite profile of the two groups (Figure A2). Considering that tumor MV constitute only a minor part of the whole plasma microvesiculome, potentially “diluting” tumor-specific findings, this was not unexpected. In the next step, we therefore aimed at identifying differentially expressed single compounds within the total MV metabolome. Here, a significantly different concentration (Figure 2a and Figure A3, Table 3) was observed for eight compounds: PC ae C40:6, lysoPC a C26:0, PC aa C38:5, PC ae C40:2, PC ae C34:2, PC ae C32:2, PC ae C38:3 and SM (OH) C16:1.

Receiver operator characteristic (ROC) curve analysis revealed that single metabolites were not suitable to discriminate between the cancer and control samples (AUC < 0.80). However, when combining seven out of the eight metabolites—PC ae C40:6, lysoPC a C26:0, PC aa C38:5, PC ae C34:2, PC ae C32:2, PC ae C38:3 and SM (OH) C16:1—for multiple logistic regression, we reached an AUC of 0.78 (95% CI [0.690;0.876]) (Figure 2b). In summary, we were thus able to identify a profile of lipid metabolites specific for MV from breast cancer patients. Comparing these eight differentially expressed metabolites in patients’ MV to metabolites detected by mass spectrometry in breast cancer tissue [13,14,27], we observed one common lipid: PC aa C38:5. There was no overlap with the lipids previously detected by Diáz-Beltrán et al. [11] in blood plasma. The list of all metabolites is depicted in Table A4.

### 2.4. LysoPC a C26:0 and PC aa C38:5 Levels Are Prognostic for Overall Survival

**Figure 3:**
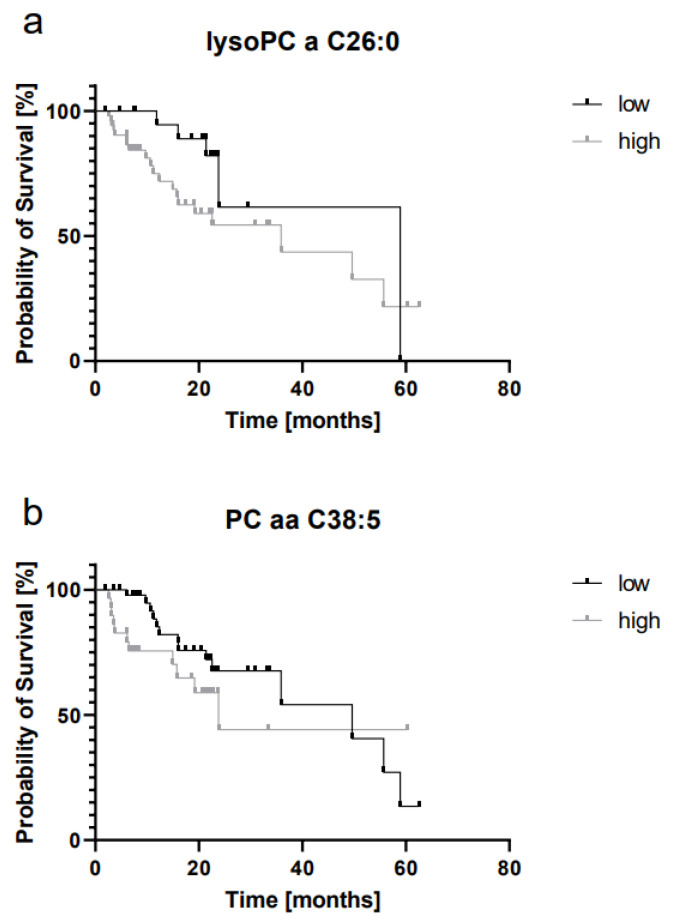
MV lipids are prognostic markers for overall survival in breast cancer. Kaplan–Meier curves (**a**,**b**): patients with high concentrations of lysoPC a C26:0 and PC aa C38:5 show a shorter OS (*p* ≤ 0.05, Gehan–Breslow–Wilcoxon test).

We then asked whether the abovementioned metabolites are correlated with the clinical outcome. The median overall survival (OS) for all patients was 15.35 (min: 1.87; max: 62.56) months. To define the optimal cut-off for the investigated metabolites, we used the freeware X-tile [28]. Patients were categorized in groups of either “high” or “low” metabolite concentration. An overview over grouping and cut-offs is presented in Table 4. Out of the eight metabolites described above, two showed a significant association with OS: elevated concentrations of both lysoPC a C26:0 and PC aa C38:5 were significantly correlated with shorter survival (Figure 3a,b).

Breast cancer patients with higher concentrations of PC aa C38:5 had a median OS of 23.80 months, whereas those with lower concentrations showed a median OS of 49.61 months (*p* = 0.017). The corresponding HR was 1.79 (95% CI [0.77;4.196]). Similarly, OS in patients with higher concentrations of lysoPC a C26:0 was significantly shorter than in the group with lower concentrations (*p* = 0.020; HR 2.07 (95% CI [0.91;4.71]), 35.90 versus 58.92 months). There was a trend towards shorter OS in patients with high concentrations of PC ae C40:6, but which did not reach statistical significance (*p* = 0.077; HR 1.70 (95% CI [0.77;3.76])).

### 2.5. The Whole Blood MV Metabolome Differentiates between Molecular Breast Cancer Subtypes

**Figure A4:**
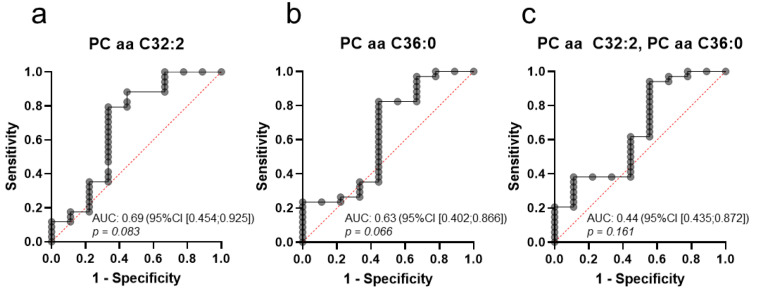
ROC-curves of significant metabolites (t-statistics) when comparing molecular subtype luminal B to basal-like breast cancer: (**a**) ROC of PC aa C32:2; (**b**) ROC of PC aa C36:0; and (**c**) combined graph (multiple logistic regression).

Molecular subtyping is crucial in breast cancer, since the molecular subclasses determine prognosis and therapy [29]. We therefore evaluated the obtained metabolomic profiles in correspondence to the respective molecular subtype (luminal A, luminal B, Her2-enriched or basal-like). Comparison of Her2-enriched to luminal A or luminal B samples, revealed significant differences in both cases (Table A2). However, the FDR was >0.90, thus not corroborating the statistical significance of these differences. Similarly, the comparison of Her2-enriched with basal-like samples or basal-like with luminal A samples did not yield any significant results. Analysis of basal-like versus luminal B yielded two significant metabolites—PC aa C32:2 and PC aa C36:0 (Table A2). However, single and combined ROC curve analysis (Figure A4) showed that both metabolites were not able to discriminate between the subtypes with sufficient statistical power. The size of the Her2-enriched and basal-like cohorts was relatively small (see also Section 3) and the numbers required to reach a power level of 0.8 as well as a significance level of 0.05 were not reached. Thus, more patient samples would be needed to confirm the detected tendencies.

**Figure 4:**
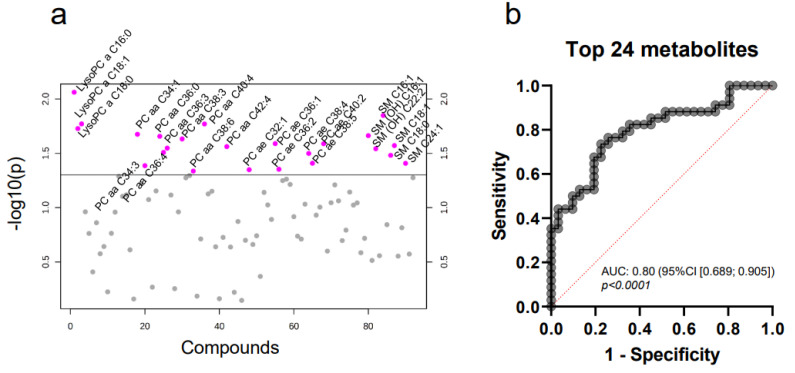
MV-based metabolomics allows the differentiation between molecular breast cancer subtypes. The metabolomic profiles of plasma MV from luminal A and luminal B breast cancer patients were compared by targeted mass spectrometry. t-statistics (**a**) reveal 24 significantly differentially expressed metabolites (unpaired Student’s t-test, relevant metabolites colored in pink). Multiple logistic regression (**b**) for combined ROC analysis of all 24 metabolites.

We then focused on patients with luminal A and B breast cancer. Both luminal A and B represented the largest groups of molecular subtypes in our cohort and had been recruited in sufficiently large numbers (31 and 34 patients, respectively) according to the a priori power analysis. The comparative analysis of these two molecular subtypes revealed 24 significantly differently expressed metabolites (Figure 4a, Table A2).

ROC curve analysis after multiple regression yielded an AUC of 0.80 (95% CI (0.689;0.905)) with a sensitivity of 73.53% and specificity of 77.42% (Figure 4b). Hence, the combination of these 24 compounds is able to reliably differentiate between luminal A and B subtypes.

### 2.6. A Distinct Metabolic Profile Discriminates between Subtype Luminal B and Healthy Controls

**Figure 5:**
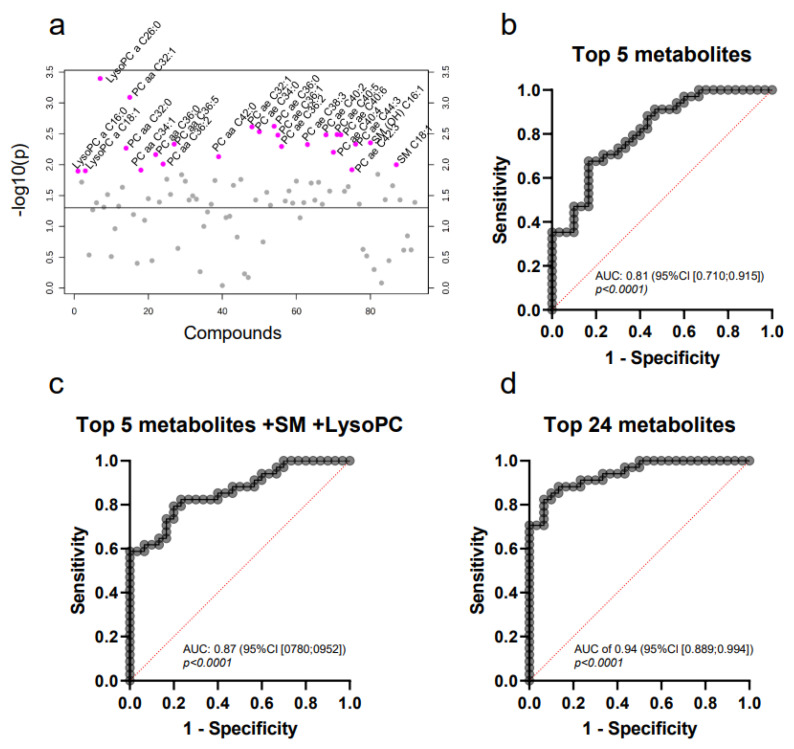
Metabolomic profiling of MV in breast cancer patients of the molecular subtype luminal B compared to healthy controls. T-statistics (**a**) reveal 24 significantly differentially expressed metabolites (unpaired Student’s t-test, relevant metabolites colored in pink). Multiple logistic regression (**b**) for the top five metabolites; (**c**) ROC combined for lysophosphatidylcholines, sphingomyelines and PC aa C32:1, PC ae C36:0 and PC ae C34:0; and (**d**) combined ROC of all 24 metabolites.

We then compared the MV metabolome of the various molecular subtypes with that of the healthy controls. Again, there was no significant difference between the small cohort of basal-like cancers and controls, most likely due to the different size of the two groups. Of note, the lysoPC a C26:0, which had already been part of the cancer-specific MV profile described above, was confirmed to be differentially expressed in the other three breast cancer subtypes compared to the controls (Table A3). Surprisingly, only in the luminal B subtype 24 metabolites were identified with a significant FDR ≤ 0.05 despite an equally large sample size in the luminal A subgroup (Figure 5a, Table A3). This suggests that luminal B patients display a particularly distinct plasma MV metabolome. In contrast to luminal B, luminal A represents the most favorable breast cancer subtype with the highest similarity to the original ductal tissue [30].

This may explain the more prominent differences found in the luminal B subtype. Therefore, we concentrated on the differences between this particular subtype and the controls.

ROC curve analysis for each of the top 24 metabolites in luminal B patients revealed that PC aa C32:1 was the best discriminator between controls and patients. The AUC of 0.79 (95% CI [0.672;0.898]) as well as the sensitivity (67.65%) and specificity (73.33%) indicated that these results are not sufficiently conclusive to support a clinical relevance. To improve the discriminatory power, we performed a combined analysis of multiple metabolites. Multiple logistic regression of the top five metabolites (lysoPC a C26:0, PC aa C32:1, PC ae C32:1, PC ae C36:0 and PC ae C34:0) (Figure 5b) yielded an enhanced AUC of 0.81 (95% CI [0.710;0.915], *p* < 0.0001), without significantly improving the sensitivity and specificity (73.53% and 70.00%, respectively). When combining PC aa C32:1, PC ae C36:0 and PC ae C34:0 with the lysoPCs and sphingomyelins, we reached an AUC of 0.86 (95% CI [0.770;0.949], *p* < 0.0001, sensitivity = 79.41%, specificity = 76.67%, Figure 5c). The best results were achieved upon combination of all 24 metabolites, with an AUC of 0.94 (95% CI [0.889;0.994], *p* < 0.0001) as well as high sensitivity (85.29%) and specificity (90.00%) (Figure 5d).

Comparing these 24 differentially expressed metabolites in patients’ MV to metabolites detected by mass spectrometry in breast cancer tissue [13,14,27], we observed again only one common lipid: PC aa C32:1. We detected LysoPC a C16:0 as the only lipid in common with the lipids previously reported to be found in blood plasma [11]. The list of all metabolites is depicted in Table A4.

### 2.7. Pathway Analysis Reveals Alterations in Glycerophospholipid and Ether Lipid Metabolism as Well as Linoleic Acid Metabolism

**Figure 6:**
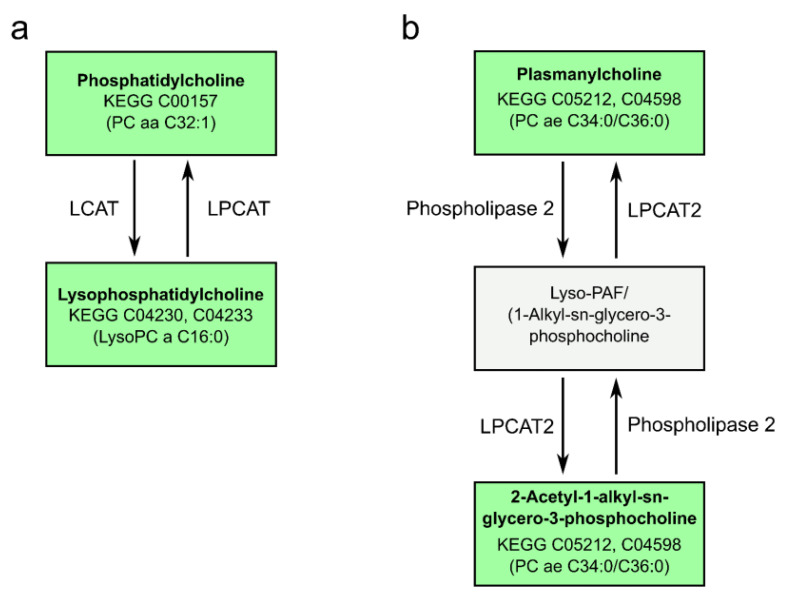
Schematic depiction of pathways detected in plasma MV by pathway analysis. Glycerophospholipid metabolism (**a**): relevant metabolites detected by metabolomics were involved in the enzymatic reaction catalyzed by lecithin-cholesterin acyl transferase (LCAT) and lysophosphatidylcholine acyltransferase (LPCAT).Changes in (**a**) were detected comparing the controls to all breast cancer patients and to the subgroup luminal B. Ether lipid metabolism (**b**): pathway analysis suggested involvement in the reaction catalyzed by LPCAT or phospholipase 2 when comparing metabolites of the control group to subgroup luminal B.

With the relevant metabolites at hand, which were either able to differentiate between the controls and breast cancer patients or between controls and the luminal B subtype, we used these to identify the underlying metabolic pathways. When comparing the controls to all breast cancer patients, alterations in the metabolites in the glycerophospholipid metabolism were found. For the latter an involvement of lecithin-cholesterin acyl transferase (LCAT) was described in colorectal cancer [31]. There were also relevant alterations in the linoleic acid and alpha-linolenic acid metabolism. The same analysis was performed with the metabolites that were different between the molecular subtype luminal B and the controls. This analysis yielded similar results. Again, as expected, we observed changes in the glycerophospholipid pathway (Figure 6a) and in the ether lipid metabolism (Figure 6b).

Additionally, alterations in the linoleic acid metabolism were proposed. Summing up, we observed relevant metabolic changes both in glycerophospholipid and ether lipid metabolism, which have been described to be associated with cancer metabolism also by other authors [31,32,33,34].

## 3. Discussion

Reprogramming of cellular energy metabolism is one of the hallmarks of cancer [35]. Changes in cancer metabolism should be reflected in the metabolite composition of cancer tissue or blood samples of patients, which has been shown for several cancer entities [36,37,38,39]. However, metabolic profiling of body fluids such as plasma is challenging since the metabolite content is influenced by factors such as diet, microbiome and exposome [40], and, in particular, by the stability of the compounds in an aqueous solution. Identifying (onco) metabolites in the more sheltered matrix of EV, which originate at least in part directly from the tumor cells, therefore seems enticing. Additionally, since EV from breast cancer patients have been shown to be critical for conferring invasiveness [8], it can be assumed that the most important compounds for these effects are associated with EV. Nevertheless, surprisingly few studies [20,21,22,41] have focused on the metabolome of EV in cancer patients, most of them characterizing either EV as a whole or exclusively sEV. In contrast, our analysis was performed on the larger MV, which confer the crucial advantage of rapid and simple harvest from peripheral blood [25].

Since we were interested in a high-throughput tool, the first question we asked was whether the AbsoluteIDQ^®^ p180 kit (Biocrates) was suitable for this matrix. Our study is the first to demonstrate the application of the AbsoluteIDQ^®^ p180 kit (Biocrates, Innsbruck, Austria) for analyzing MV. Validation by parallel analysis via an untargeted metabolomics approach on a QToF mass spectrometer showed that most of the significant metabolites were also detected with the second method. Although the kit detected a broad variety of compounds, we found mostly lipids, such as (lyso)PCs and SMs, as the predominant metabolites. This is not surprising, since these compounds represent key players in energy housekeeping, membrane structure and signal transduction [42,43] and are to be expected in high abundance in the large membrane-enclosed MV. In contrast, the absence of other metabolites that are highly abundant in blood plasma, e.g., amino acids and carnitines, underlines the quality of our preparation protocol for EV. However, some metabolites of low abundance, but nevertheless of functional importance, may have been missed using the standardized approach, suggesting that the application of specifically optimized mass spectrometry methods would be necessary.

Through metabolic profiling of blood plasma several authors have previously identified cancer-specific metabolites that differentiate between healthy subjects and lung [44,45,46] or breast cancer patients [11,12]. Now, we demonstrate for the first time that the metabolome of blood-derived MV from breast cancer patients differs significantly from that of healthy controls. We describe eight lipids that are present in higher concentrations in cancer patients. The profile in our study is partially superimposable with the metabolic profile identified by Qiu et al. [12] in plasma samples of breast cancer patients (6/8 metabolites). Likewise, 11/24 metabolites in the cohort luminal B vs. controls were identical with the Qiu data. This is hardly surprising, given that plasma contains EV in addition to soluble molecules. Depending on the sample preparation before mass spectrometry, EV can also be lysed and included in subsequent analyses. The fact that we observed higher lipid concentrations of all metabolites than Qiu et al. is in line with this assumption.

Some of the identified compounds were not only suitable discriminators between cancer patients and controls, but also markers of poor clinical outcome. Higher concentrations of the phosphatidylcholine (PC) “PC aa C38:5” as well as the lysoPC “lysoPC a C26:0” were associated with significantly shorter OS, thus underlining the prognostic relevance of this finding. In accordance, Guo and coworkers had previously described that deviations of lysoPCs were associated with cancer progression [44]. Interestingly, this association differs between cancer entities. Prostate cancer patients showing high amounts of lysoPCs before the onset of disease seem to have a lower risk for advanced disease at the time of diagnosis [47].

Comparison of the MV metabolome from breast cancer patients with different molecular subtypes revealed distinct features predominantly for the two large groups of luminal A and B versus each other as well as luminal B versus controls. Despite some variations regarding single metabolites, we could not detect significantly different profiles for the Her2-enriched and the basal-like cohort, although their diverging biological characteristics would strongly suggest such differences. However, both subcohorts comprised only few patients (basal-like: 9 patients; Her2-enriched: 4 patients); thus, the statistical power to detect significant differences was not reached. The reason for the small numbers was that only a minority of the patients in these clinically aggressive subgroups could be included in our study, since most of them were under cancer-specific (chemo)therapy that had been established as an exclusion criterion to avoid contamination with apoptotic bodies.

There are only limited data on the breast cancer metabolome in the literature and most of these are derived from tissue analysis. In accordance with our results, Ide and coworkers [14] described an elevated content of saturated and unsaturated PC in breast cancer tissue, in particular PC aa C32:1, PC aa C34:1 and PC aa C36:0, which were significant metabolites in the luminal B subtype profile we observed. Similarly, PC aa C32:1 was found by other authors in the tissues of breast cancer and oral squamous carcinoma [13,27]. Only two recent studies aimed to identify metabolome signatures specific for the various molecular subtypes by analyzing blood plasma [11,48]. Similar to our data, Dìaz-Béltran et al. found several candidates that were able to discriminate between the subtypes; however, there is no overlap between our profile for luminal B and the one proposed by that study. Fan et al. were able to distinguish between molecular subtypes using eight metabolites. However, none of these metabolites overlapped with our data. A distinct classification of molecular subtypes may explain these findings, in addition to the fact that our source was MV rather than plasma. On a second note, we identified several lipids not previously described by metabolic studies investigating tissue samples. This is to be expected, since MV extracted from the blood represent a mixture of MV from platelets, red blood, endothelial and immune cells, with only a minority of tumor MV. Cancer cells and their MV influence the microenvironment by reprogramming the innate and adaptive immune cells [49,50]. Thus, our approach offers the advantage to detect specific metabolites not only in the tumor MV but also to characterize metabolic changes in the whole blood MV “reactome”, allowing a broader, holistic view on cancer metabolism.

Lipids are critically involved in cancer-related signaling cascades. Accordingly, pathway analysis of our detected metabolites yielded two significantly altered metabolic pathways. Distinct changes in the glycerophospholipid metabolism were found in breast cancer MV samples in comparison to healthy controls as well as in the luminal B subtype MV versus controls. Within this pathway, particularly the conversion of the PC with the KEGG identifier C00157 (e.g., PC aa C32:1) into lysoPCs with the KEGG identifiers C04230 and C04233 (e.g., lysoPC a C16:0) and back were involved. These reactions are catalyzed by the enzymes lecithin–cholesterol acyltransferase (LCAT) and lysophosphatidylcholine acyltransferase (LPCAT), respectively. An accumulation of PC (16:0/16:1), a potential isomer of PC aa C32:1, and the dysregulation of the responsible enzyme has also been observed by others in colorectal cancer tissues [31]. The second significantly involved pathway, the ether lipid metabolism, did not discriminate between the whole cohort of cancer patients and the controls but predominantly between the luminal B subtype and the controls. The proposed relevant metabolites were the KEGG identifiers C05212 and C04598, which comprise the compounds PC ae C36:0 and PC ae C34:0 found in our analyses. These isomers are substrates for phospholipases and the enzyme LPCAT2. Changes in ether lipid metabolism have recently been described on the genomic and metabolic level in vitro as well as in vivo for breast cancer patients [32,33,34]. There are first pre-clinical efforts to target ether lipid metabolism in cancer cells [51].

Taken together, we demonstrate that metabolomic profiling of blood-derived MV is feasible using a standardized high-throughput tool for mass spectrometry (AbsoluteIDQ^®^ p180 kit; Biocrates, Innsbruck, Austria). Analysis of MV and other vesicles, in contrast to whole plasma, is of particular interest for the understanding of breast cancer progression, since we have shown earlier that vesicles, and not soluble plasma compounds, mediate the invasiveness of breast cancer cells [8]. The identified lipid biomarkers did not only discriminate between breast cancer patients and controls but were also prognostic markers of clinical outcome. Additionally, a distinct metabolic profile was described for the luminal B subtype, which was very different from the controls and the luminal A cohort. This is in line with the biological behavior of these subtypes, the luminal A group representing the most favorable variant with the highest similarities to the original ductal tissue [30]. Assumedly, the aggressive Her2-positive and triple-negative subtypes display distinct metabolic features but our subcohorts were too small for statistically reliable conclusions.

In summary, metabolic profiling of MV yields promising biomarkers for diagnosis and prognosis of breast cancer. Further studies are necessary to determine whether they are indeed of functional relevance and to clarify their potential mode of action.

## 4. Materials and Methods

### 4.1. Patient Recruitment and Data Extraction

This project was approved by the local ethics committee (project number 3/2/14). Sample size was calculated a priori to reach a power of 0.8, an alpha-error of 0.05 and an effect size of 0.8. Case allocation was 2:1 for breast cancer patients versus healthy controls (N (breast cancer patient) = 39; N (healthy control) = 19) and 1:1 for any sub-group analysis (N (per subgroup) = 26).

Female breast cancer patients (*n* = 78) and controls (*n* = 30) were recruited consecutively between March 2014 and June 2020. Inclusion criteria were advanced primary tumor or metastasized disease. To avoid contamination with apoptotic bodies as far as possible, we only included patients without or prior to chemo-/radiotherapy and, rarely, with stable disease under anti-hormonal therapy. Patients were recruited at the department for Hematology and Medical Oncology, the department of Gynecology and Obstetrics (both University Medical Center Goettingen) and an associated out-patient clinic for medical oncology. Female controls were recruited from the medical staff of the institute, healthy volunteers and regular blood donors. Blood samples were drawn after written consent. Histology and molecular breast cancer subtypes had been evaluated by the Department of Pathology, University Medical Center Goettingen. Patients were defined as oligometastatic if they had ≤3 metastasis. Disease status—stable vs. progressive disease—was assessed at the time of recruitment. Patient characteristics are summarized in Table 1.

### 4.2. Sample Preparation and Mass Spectrometry

MV were isolated from 5 to 15 mL EDTA-anticoagulated peripheral blood from healthy controls and breast cancer patients. In order to prevent plasma degradation and metabolite leakage, samples were processed immediately (<2 h) after collection. Blood plasma was obtained by centrifuging the samples for 15 min at 1200× *g*. MV were then purified by two additional centrifugation steps at 1500× *g* (15 min) and at 14,000× *g* (35 min). The 14,000× *g* pellets containing the MV were washed and resuspended in PBS, as previously described [8,25]. Samples were quantified before storage at −20 °C by measurement of the protein content with the Lowry assay (Dc protein assay, Bio-Rad, Thermo Fisher, Bremen, Germany). For mass spectrometry, MV (50 µg protein) were cracked by adding 30 µL methanol/water (9:1) and freezing/thawing samples three times at −150 °C.

Targeted metabolomic analysis was performed using the AbsoluteIDQ p180-Kit (Biocrates Lifesciences, Innsbruck, Austria) applied to a BEH Amide column (Waters Cooperation, Milford, MA, USA) and a Xevo TQ-S mass spectrometer (Waters Cooperation, Milford, MA, USA). The kit was processed using the guidelines of the manufacturer. Measurement of the kit consisted of two separate analyses, flow injection analysis (FIA-MS) for lipids and liquid chromatography MS (LC-MS) for amines. FIA-MS was used for the quantification of up to 145 metabolites (including 40 acylcarnitines, 15 sphingolipids, 76 PC and 15 lysoPC). LC-MS analysis includes the measurement of 21 canonical amino acids and 21 other biogenic amines, such as neurotransmitters.

Briefly, 30 μL of sample were applied to the kit plate in 2 separate steps of 10 and 20 µL each and dried using a positive-pressure manifold (Waters Cooperation, Milford, MA, USA). Consecutively, the dried sample plate was treated with phenylisothiocyanate to derivatize amines and dried under nitrogen flow. Samples from the plate were extracted using 5 mM ammonium acetate in methanol and diluted in flow injection analysis solvent for FIA analysis and in water for LC-MS analysis, respectively. After the FIA and LC-MS runs, LC-MS data were first analyzed using MassLynx V4.1 software (Waters Cooperation, Milford, MA, USA) using the supplied analysis method by Biocrates, confirmed manually, and imported into the software supplied by the kit (MetIDQ Carbon, Biocrates Lifesciences, Innsbruck, Austria). FIA data were directly loaded into MetIDQ Biocrates Lifesciences, Innsbruck, Austria and interpreted automatically.

The analytical quality of each kit run was validated according to the guidelines of the manufacturer. Briefly, three different concentrations of plasma-based quality controls were measured. The medium concentration quality control sample was distributed 4 times across the plate to validate the quality throughout the entire run. Quality control results were assessed automatically based on internal criteria via the MetIDQ software (Biocrates Lifesciences, Innsbruck, Austria). The untargeted mass spectrometry metabolomics approach (UPLC-MSMS) was conducted on an Acquity UPLC^®^ I-Class (Waters Cooperation, Milford, MA, USA) coupled to a Xevo G2S (Waters Cooperation, Milford, MA, USA) equipped with an electrospray ionization (ESI) ion source. An Acquity UPLC^®^ HSS T3 1.8 µm 2.1 × 100 mm column (Waters Cooperation, Milford, MA, USA) was used for chromatographic separation of the compounds. The composition of the mobile phase A and B was as following: 10 mM ammonium formate, 0.1% formic acid, 40% H_2_O, 60% acetonitrile (A) and 10 mM ammonium formate, 0.1% formic acid, 10% acetonitrile, 90% 2-propanol (B); 2 µL of the sample were injected. Phase B was set at 35% and subsequently increased to 90% over 55 min, before the column was equilibrated for another 5 min under the initial conditions. The flow rate was 0.2 mL/min. The column temperature was set to 55 °C. All compounds were analyzed in positive mode with a capillary voltage of 2.4 kV and source temperature of 120 °C. The mass range setting was *m/z* 70 to 1000. For each setting, 3–4 technical replicates were performed. Alignment of the spectra and peak picking were processed using Progenesis QI software (Non-linear Dynamics, Wilmslow UK) and the open software http://metaboanalyst.ca (version 4.0 [52]).

### 4.3. Nanoparticle Tracking Analysis (NTA)

Size distribution and concentration of MV were analyzed on a Nanosight NS300 (Malvern Panalytical Ltd., Malvern, UK) equipped with a 488 nm laser and a sCMOS camera. MV samples equivalent to 50 µg protein were diluted in PBS up to a final volume of 400 µL to obtain a concentration of 20–60 particles per frame. At least three videos of 60 s were recorded for each sample. Temperature setting was controlled at 25 °C and syringe pump flow rate at 50 MV of the breast cancer cell line SK-BR-3 were used as positive size controls. The NTA 2.3 software (build 013) was used for analyzing videos and calculating mean values of particle concentration and size of each sample.

### 4.4. Western Blot

Human MFC-7 cells were cultured in RPMI-1640 medium supplemented with 10% fetal calf serum (FCS). Protein expression of cell lysate, human plasma and patient MV was assessed using the following primary antibodies: α-ACTININ-4 (#sc-390205), RGAP1 (#sc-271110), β-AKTIN (#sc-47778), APOA1 (#sc-376818, all from Santa Cruz Biotechnology). The HRP-linked secondary antibody was obtained from cell signaling (#7076). In total, 17 µg of protein were subjected to SDS-PAGE and subsequently blotted onto nitrocellulose. Membranes were blocked in TBST (137 mM NaCl, 20 mM Tris pH 7.6, 0.1% (*v*/*v*) Tween-20) before incubation with the respective primary antibody overnight at 4 °C. After three washing steps for 5 min each in TBST, the membranes were incubated with the secondary antibody at RT followed again by three 5 min washing steps in TBST. Signals were detected with ECL Prime (GE Healthcare, Chicago, IL, USA) on a LAS-40,000 imager (Fujifilm, Düsseldorf, Germany). Ponceau staining was routinely used as the loading control.

### 4.5. Statistical Analysis and Bioinformatics

The following modules of the online freeware Metaboanalyst (version 4.0 [52]) were used for analysis: “Statistical Analysis”, “Pathway Analysis” and “Biomarker Analysis”. The t-test was used for identifying significant metabolites differing in concentration/peak between groups. A *p*-value of ≤0.05 was considered significant. In that case, the metabolite was included into further analysis. The false discovery rate (FDR) was obtained for each metabolite analyzed by t-statistics. The principal component analysis (PCA) and heatmapping tool of Metaboanalyst were also applied.

ROC curves for multiple metabolites were calculated by multiple logistic regression using GraphPad Prism (Version 8.4.2, Graphpad Holdings, San Diego, CA, USA). X-tile (Version 3.6, New Haven, CT, USA) was used as bioinformatics tool to assess the optimal cut-off for metabolite concentrations, dividing samples into groups of “high” and “low” metabolite concentrations [28]. We used GraphPad Prism (Version 8.4.2, Graphpad Holdings, San Diego, CA, USA) for survival analysis. The endpoint of the survival analysis was death or loss in follow-up. The Gehan–Breslow–Wilcoxon test was used for calculating the Kaplan–Meier curves, and a *p*-value < 0.05 was considered significant. Mantel–Haenszel analysis was applied for calculating the hazards ratios (HR).

Pathway analysis was performed for significant metabolites. The list of metabolites recognized by the Biocrates AbsoluteIDQ^®^ p180 kit (Biocrates Lifesciences, Innsbruck, Austria) are listed at http://metap.helmholtz-muenchen.de/metap2/metabolites/Details/, refer to [53]. All proposed KEGG IDs [54] were used for pathways analysis using the “Pathway Analysis” module of Metaboanalyst.

## Figures and Tables

**Table 1:** Clinical characteristics of the patients and healthy controls.

Clinical Characteristics	
Age median (patients, min-max range) Age median (healthy controls, min-max range)	63 (27–92) 39 (21–65)
**Histological subtype**	
Invasive ductal	49
Invasive lobular	14
Invasive ductal/lobular	1
Other	3
Not defined	11
**Molecular subtype**	
Luminal A	31
Luminal B	34
Basal-like	9
Her2-enriched	4
**Metastasis**	
Oligometastasis (≤3 metastasis)	18
Polymetastasis (>3 metastasis)	50
**Location of metastasis**	
Bone	48
Brain	15
Lung	25
Other	33
**Stable vs. progressive disease**	
Progressive disease	29
Stable disease	49
**Metabolic comorbidities of patients**	
Obesity	14/76 *
Diabetes mellitus	8/77 **
Lipometabolic disorders	6/77 **

Note: *, data for two; and **, one case not available.

**Table 2:** Metabolites identified in both targeted and untargeted mass spectrometry approaches.

Lysophosphatidylcholines
LysoPC a C16:0, LysoPC a C18:0, Lyso PC a C18:1, LysoPC a C18:2, LysoPC a C20:4, LysoPC a C24:0
**Sphingomyelins**
SM (OH) C14:1, SM (OH) C22:1, SM(OH) C22:2, SM (OH) C24:1, SM C18:0, SM C18:1, SM C20:2, SM C24:0, SM C26:1

**Table 3:** T-statistics revealed eight significant metabolites differentiating breast cancer patients from the controls.

Metabolite	t-Value	*p*-Value	FDR
PC ae C40:6	−3.8915	0.0002	0.016
lysoPC a C26:0	−2.6967	0.0081	0.3277
PC aa C38:5	−2.5989	0.0107	0.3277
PC ae C40:2	−2.2459	0.0268	0.3946
PC ae C34:2	−2.2170	0.0288	0.3946
PC ae C32:2	−2.1730	0.0320	0.3946
PC ae C38:3	−2.1701	0.0322	0.3946
SM (OH) C16:1	−2.1441	0.0343	0.3946

**Table 4:** Cut-off for categories of high or low biomarker concentration in patients with breast cancer.

Metabolite	Group	N (Patients)	N (Events)	Cut-Off (µmol/L)
lysoPC a C26:0	low	26	5	0.03
	high	52	30	
PC aa C38:5	low	49	13	2.56
	high	29	9	
PC ae C32:2	low	33	8	0.03
	high	45	17	
PC ae C34:2	low	24	6	0.39
	high	54	19	
PC ae C38:3	low	19	6	0:27
	high	59	19	
PC ae C40:2	low	56	18	0.18
	high	22	7	
PC ae C40:6	low	39	10	0.14
	high	39	15	
SM (OH) C16:1	low	49	16	0.27
	high	29	9	

## Data Availability

The authors confirm that the data supporting the findings of this study are available within the article and its Appendix A.

## Appendix A

**Table A1:** Lipid classes and single lipids detected in EV samples using the Biocrates IDQ^®^ p180 kit (Biocrates Lifesciences, Innsbruck, Austria).

Metabolite Class	Metabolites Detected
**LysoPC**	lysoPC a C16:0 lysoPC a C18:0 lysoPC a C18:1 lysoPC a C18:2 lysoPC a C20:4 lysoPC a C24:0 lysoPC a C26:0 lysoPC a C26:1 lysoPC a C28:0 lysoPC a C28:1
**PC aa**	PC aa C24:0 PC aa C28:1 PC aa C30:0 PC aa C32:0 PC aa C32:1 PC aa C32:2 PC aa C32:3 PC aa C34:1 PC aa C34:2 PC aa C34:3 PC aa C34:4 PC aa C36:0 PC aa C36:1 PC aa C36:2 PC aa C36:3 PC aa C36:4 PC aa C36:5 PC aa C36:6 PC aa C38:0 PC aa C38:3 PC aa C38:4 PC aa C38:5 PC aa C38:6 PC aa C40:2 PC aa C40:3 PC aa C40:4 PC aa C40:5 PC aa C40:6 PC aa C42:0 PC aa C42:1 PC aa C42:2 PC aa C42:4 PC aa C42:5 PC aa C42:6
**PC ae**	PC ae C30:0 PC ae C30:1 PC ae C30:2 PC ae C32:1 PC ae C32:2 PC ae C34:0 PC ae C34:1 PC ae C34:2 PC ae C34:3 PC ae C36:0 PC ae C36:1 PC ae C36:2 PC ae C36:3 PC ae C36:4 PC ae C36:5 PC ae C38:0 PC ae C38:1 PC ae C38:2 PC ae C38:3 PC ae C38:4 PC ae C38:5 PC ae C38:6 PC ae C40:1 PC ae C40:2 PC ae C40:3 PC ae C40:4 PC ae C40:5 PC ae C40:6 PC ae C42:1 PC ae C42:2 PC ae C42:3 PC ae C44:3 PC ae C44:5 PC ae C44:6
**SM**	SM C16:0 SM C16:1 SM C18:0 SM C18:1 SM C20:2 SM C24:0 SM C24:1 SM C26:0 SM C26:1
**SM (OH)**	SM (OH) C14:1 SM (OH) C16:1 SM (OH) C22:1 SM (OH) C22:2 SM (OH) C24:1

**Table A2:** Analysis of molecular subtypes tested against each other.

	Metabolite	t-Value	*p*-Value	FDR
***Her2-enriched*** ***Luminal A***	PC aa C24:0	2.3912	0.02266	0.9786
***Her2-enriched*** ***Luminal B***	PC ae C40:4	−2.0812	0.0446	0.9456
***Basal-like*** ***Luminal B***	PC aa C32:2	−2.6532	0.0113	0.3688
	PC aa C36:0	−2.5427	0.0149	0.3688
***Luminal A*** ***Luminal B***	lysoPC a C16:0	−2.7094	0.0087	0.1687
	SM C16:0	−2.5227	0.0142	0.1687
	lysoPC a C18:1	−2.4528	0.0170	0.1687
	PC aa C40:4	−2.4522	0.0170	0.1687
	lysoPC a C18:0	−2.4133	0.0187	0.1687
	PC aa C34:1	−2.3647	0.0211	0.1687
	SM OH C16:1	−2.3531	0.0218	0.1687
	PC aa C36:2	−2.3475	0.0220	0.1687
	PC aa C38:3	−2.3247	0.0233	0.1687
	PC ae C36:1	−2.2838	0.0258	0.1687
	PC ae C40:2	−2.2831	0.0258	0.1687
	SM C18:1	−2.2667	0.0269	0.1687
	PC aa C42:4	−2.2577	0.0274	0.1687
	PC aa C36:4	−2.2442	0.0283	0.1687
	SM OH C22:2	−2.2387	0.0287	0.1687
	PC aa C36:3	−2.2061	0.0310	0.1687
	PC ae C38:4	−2.1969	0.0317	0.1687
	SM C18:0	−2.1799	0.0330	0.1687
	PC ae C38:5	−2.1078	0.0390	0.1723
	SM C24:1	−2.1067	0.0391	0.1723
	PC aa C34:3	−2.0851	0.0411	0.1723
	PC ae C36:2	−2.0515	0.0444	0.1723
	PC ae C32:1	−2.0474	0.0448	0.1723
	PC aa C38:6	−2.0347	0.0461	0.1723

**Table A3:** Differentially expressed metabolites, comparing molecular subtypes to healthy controls. Due to the abundance of significant metabolites for luminal B only those with an FDR ≤ 0.05 are shown here.

	Metabolite	t-Value	*p*-Value	FDR
***Luminal A***	lysoPC a C26:0	−2.0632	0.0435	0.8630
***Luminal B***	lysoPC a C26:0	−3.7419	0.0004	0.0332
	PC aa C32:1	−3.5215	0.0008	0.0332
	PC ae C36:0	−3.1676	0.0024	0.0332
	PC ae C32:1	−3.1676	0.0024	0.0332
	PC ae C34:0	−3.0978	0.0029	0.0332
	PC ae C40:5	−3.0619	0.0033	0.0332
	PC ae C40:6	−3.0584	0.0033	0.0332
	PC ae C40:2	−3.0581	0.0033	0.0332
	PC ae C36:1	−3.0542	0.0033	0.0332
	SM OH C16:1	−2.9544	0.0044	0.0332
	PC aa C36:5	−2.9370	0.0046	0.0332
	PC ae C44:3	−2.9368	0.0046	0.0332
	PC ae C38:3	−2.9318	0.0047	0.0332
	PC ae C36:2	−2.9057	0.0051	0.0332
	PC aa C32:0	−2.8831	0.0054	0.0332
	PC ae C40:4	−2.8284	0.0063	0.0362
	PC aa C36:0	−2.7971	0.0069	0.0371
	PC aa C42:0	−2.7682	0.0074	0.0379
	PC aa C36:2	−2.6660	0.0098	0.0461
	SM C18:1	−2.6564	0.0100	0.0461
	PC ae C42:3	−2.5854	0.0121	0.0487
	PC aa C34:1	−2.5802	0.0123	0.0487
	lysoPC a C18:1	−2.5700	0.0126	0.0487
	lysoPC a C16:0	−2.5666	0.0127	0.0487
***Her2-enriched***	lysoPC a C26:0	−3.1351	0.0037	0.3374

**Table A4:** Differentially expressed metabolites of all breast cancer patients and of the luminal B subgroup compared to the metabolic profiles already published in the literature.

Author	Overlapping Metabolites (All Breast Cancer Patients)	Non-Overlapping Metabolites Published
**Dìaz-Béltran et al.** **(blood plasma)**	0	LyoPC a C14:0 LysoPC a C16:0 LysoPC a C23:0 Biliverdin
**Ide et al., 2013** **Uchiyama et al., 2014** **Hosokawa et al., 2017** **(tissue)**	PC aa C38:5	PC aa C36:1 PCaa C34:1 PC aa C38:6 PC aa C32:1 PC aa C34:0 PC aa C30:0
**Author**	**Overlapping metabolites** (***luminal B patients*)**	**Non-overlapping metabolites published**
**Dìaz-Béltran et al.** **(blood plasma)**	LysoPC a C16:0	LyoPC a C14:0 LysoPC a C23:0 Biliverdin
**Ide et al., 2013** **Uchiyama et al., 2014** **Hosokawa et al., 2017** **(tissue)**	PC aa C32:1	PC aa C38:6 PC aa C34:0 PC aa C30:0 PC aa C38:5 PC aa C36:1 PC aa C34:1
